# Exploring Nursing Research Culture in Clinical Practice: Qualitative Ethnographic Study

**DOI:** 10.2196/50703

**Published:** 2024-01-09

**Authors:** Hyeyoung Hwang, Jennie C De Gagne, Leeho Yoo, Miji Lee, Hye Kyung Jo, Ju-eun Kim

**Affiliations:** 1 College of Nursing Ewha Womans University Seoul Republic of Korea; 2 Adventist HealthCare Shady Grove Medical Center Rockville, MD United States; 3 School of Nursing Duke University Durham, NC United States; 4 Jeonbuk National University Hospital Jeonju Republic of Korea; 5 Health Insurance Review & Assessment Service Wonju Republic of Korea

**Keywords:** clinical nursing research, ethnography, evidence-based nursing, nursing research, qualitative research

## Abstract

**Background:**

Cultivating a positive research culture is considered the key to facilitating the utilization of research findings. In the realm of clinical nursing research, nurses conducting research may find the utilization of findings challenging due to the lack of a positive research culture.

**Objective:**

This study aims to identify and describe the sociocultural context of nursing research in a clinical setting at a Korean tertiary hospital.

**Methods:**

We included participant observation and ethnographic interviews with 6 registered nurses working in a medical-surgical unit in a Korean tertiary hospital who had experience conducting nursing research in clinical settings in this qualitative ethnographic study. The study was conducted from April 2022 to May 2022. Data analysis was conducted using Spradley’s ethnographic approach, which includes domain analysis, taxonomic analysis, componential analysis, and theme analysis, and occurred concurrently with data collection.

**Results:**

The overarching theme identified for nursing research culture in clinical practice was the development of a driving force for growth within the clinical environment. This theme encompasses (1) balancing positive and negative influences in the research process, (2) fostering transformational change for both nurses and patients, and (3) promoting complementary communication among nurses.

**Conclusions:**

Clinical research plays a vital role in nursing practice that requires a balance of supportive elements, such as patient-driven research questions and hospital research support, with practical challenges such as shift work and high work intensity. This study found that a positive clinical nursing research culture can serve as a unifying bridge, connecting researchers, patients, who serve as both the origin and ultimate beneficiaries of research, and hospitals that facilitate research endeavors. Future research should explore whether the themes derived from this study fully reflect a clinical nursing research culture comprising patients, nurses, and the hospital environment and determine what requirements are needed to establish such a nursing research culture.

## Introduction

### Overview

Nurses are increasingly expected to understand and actively participate in research endeavors and to use emerging research evidence as a foundation for their professional practice [1]. This expectation is highlighted by the International Code of Ethics for Nurses, a widely esteemed code of ethics in the nursing profession that explicitly stipulates that nurses should engage in research as an integral aspect of their profession, cultivate research-driven professional acumen, and implement evidence-based findings into their practice [2]. Similarly, the Korean Code of Ethics for Nurses underscores the responsibility of professional nurses to contribute to the development of nursing standards and the advancement of nursing research [3].

Nursing research is defined as a systematic inquiry designed to develop evidence-based information about issues important to the nursing profession, including nursing practice, education, administration, and informatics [1]. Clinical nursing research is a subset of nursing research that focuses specifically on nursing practice to promote and support patients’ health, well-being, and quality of life [1,4]. Because nurses constitute the largest group of frontline providers of health care, clinical nursing research has increasingly gained recognition as a vital path to implementing practical, efficient, and economically viable strategies that reduce hospital errors, minimize unnecessary expenditures, and enhance patient outcomes [5].

Research utilization, also referred to as knowledge translation, is a pivotal component of the clinical nursing research process; it involves the generation, distribution, and integration of research findings into clinical practice [4]. Research utilization entails not only the implementation of evidence into practice but also the continuous monitoring and evaluation of changes in practice [6]. Given their role as frontline caregivers in clinical settings, nurses are crucially responsible for translating research findings into clinical nursing practice [7]. Nurses must be motivated and prepared to synthesize the results of existing studies, apply them to clinical practice, and formulate research questions directly within the clinical setting to generate new evidence, yet nurses may remain unengaged in research activities due to a lack of capacity or support to implement research findings into their daily clinical practice [4,8].

The effective utilization of research findings relies on three essential factors: (1) fostering a positive research culture, (2) garnering interest from individuals capable of applying these findings in practice, and (3) securing comprehensive support from governmental bodies, managers, and peers [9]. This study posits that fostering a positive research culture inherently encompasses the other 2 factors because a thriving research culture naturally generates interest and encourages support to translate research findings into practice. We posit, therefore, that a positive research culture is foundational to enhancing individual research interests and garnering organizational support.

Cultivating a positive research culture is essential because research utilization can prove challenging for clinical nurses due to a lack of time, knowledge, research supervision, and support [8]. This study seeks to explore the culture of clinical nursing research in Korea to provide substantive insights for cultivating a positive research culture.

### Background

Defining culture poses a formidable challenge due to its inherent complexity; however, adopting a cultural perspective enables an understanding of why certain phenomena may occur in specific ways [10]. Consequently, to understand the essence of any phenomenon, it is necessary to explore the specific culture to which it belongs. A comprehensive understanding of clinical nursing research requires a deep familiarity with the culture of nursing research within specific clinical settings.

In the United Kingdom, because nursing functions within the National Health Service framework, government-led health care changes have seldom been research-based, and few studies have investigated the nature of clinical nursing research culture [9]. The United Kingdom has two distinct nursing subcultures: one for nurses and another for researchers, each characterized by differing values and language use [9]. Despite efforts to bridge these cultural differences, an explicit definition of a nursing research culture in clinical practice in the United Kingdom remains elusive [9]. The United Kingdom has encountered challenges in fostering a nursing research culture due to such factors as a shortage of adequately qualified research-active personnel, underdevelopment of research culture in many departments, limited dedicated research funding, and recurring competing demands on nurse academics [11].

A recent study in Denmark explored nurse researchers’ experiences in clinical roles and their perceptions of the nursing research culture in clinical practice [12]. In their case study of nurse researchers’ experiences of the presence of a nursing research culture in clinical practice, Berthelsen and Hølge-Hazelton [12] described nursing research culture as “caught between a rock and a hard place,” reflecting the dual pressures arising from a limited academic tradition among nurses and a lack of recognition from physicians. In Australia, the authors of a survey of interdisciplinary researchers concluded that an enabling research culture should comprise research productivity, positive collegial relationships, inclusivity, noncompetitiveness, and effective research processes and training [13], but notably, all participants in this study were researchers rather than clinical nurses. Given that clinical nurses are increasingly tasked with involvement in clinical nursing research [14,15], relying solely on nursing researchers to depict the entirety of the clinical nursing research culture presents inherent limitations.

In South Korea, nursing research has been active since the 1980s [16], with clinical nursing research primarily conducted at the tertiary hospital level [14,17-19]. Studies conducted in Korea have explored facilitators and barriers to nursing research in clinical practice, including clinical nurses’ knowledge and skills, acknowledgment of the importance of nursing research, organizational support, resource and facility constraints, time limitations, lack of leadership interest, challenges in statistical analysis, and the generalization of research results [14,20-22]. Although these studies have identified factors influencing the research performance of clinical nurses, the specific nature of the clinical nursing research culture in Korea remains largely unexplored.

To gain a nuanced understanding of the sociocultural context surrounding nursing research in clinical settings, it is essential to explore the culture of the nursing research environment from both observer (etic) and insider (emic) perspectives. Our theoretical framework emphasizes the central role of research utilization in clinical nursing research and has guided each step of our inquiry. In alignment with this framework, our research questions were designed to explore the interplay between the prevailing research culture and the practical utilization of research findings within clinical settings. The selection of participants, the structure of the interviews, and the focal points of our observations were carefully aligned with our framework’s emphasis on discerning the sociocultural nuances inherent to nursing research.

### Purpose

This study aims to identify and describe the sociocultural context of clinical nursing research within a Korean tertiary hospital. The guiding research questions are the following: (1) what is the sociocultural context of clinical nursing research in a Korean tertiary hospital, and how does it impact clinical nurses’ research activities? (2) How do clinical nurses perceive the research environment’s culture, and what shared values and beliefs do they hold regarding nursing research in this context? (3) What are the facilitating and hindering factors impacting clinical nurses’ research activities? Through participant observation and ethnographic interviews, we sought to uncover shared values and beliefs inherent in the visible phenomenon of the research environment culture of clinical nurses.

## Methods

### Overview

Ethnography facilitates the understanding of cultural phenomena, enabling in-depth comprehension of the subject culture from the vantage point of its native participants [23,24]. Therefore, Spradley’s [23,24] ethnographic method is aptly suited for this study as it focuses on conducting in-depth interviews with clinical nurses and gaining understanding of the context from both internal and external perspectives.

**Figure 1:**
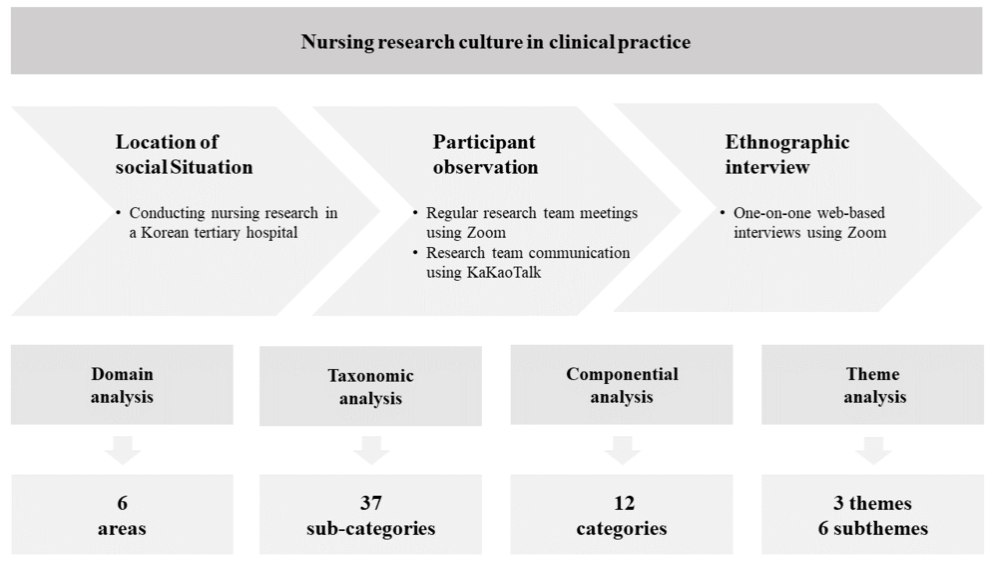
An overview of research methods.

Our analytical approach, deeply rooted in the emphasized theoretical framework, enabled us to interpret our findings in the broader context of research utilization in clinical nursing. This harmonious amalgamation of theory and method allowed us to unearth insights deeply rooted in the lived experiences of clinical nurses, illuminating the multifaceted nature of research engagement in clinical practice. By detailing the application and influence of our theoretical framework explicitly at each research stage, we aimed to provide a clearer and more comprehensive picture of how theoretical underpinnings shaped this study, addressing any potential concerns regarding the role and application of the theoretical framework in our research. An overview of the method is presented in Figure 1.

### Design

Initially, social situations were identified based on Spradley’s [23] participant observation, analyzing places, actors, and activities. Participant observation and ethnographic interviews explored and described the research environment and culture of clinical nurses. This study adhered to and was reported according to the Standard for Reporting Qualitative Research (SRQR) [25]. The result of SRQR is presented in Multimedia Appendix 1.

### Setting

In a tertiary hospital in South Korea, nurses submit clinical questions annually, and those whose questions are deemed valuable are given opportunities for advancement in clinical nursing research. In a participating medical-surgical unit of this hospital, clinical nursing research is underway that explores the following clinical question: “Is high-dose bowel preparation necessary before colonoscopy?” The research study compares bowel cleanliness, patient compliance, and side effects arising from different bowel preparations for patients undergoing colonoscopy.

Participant observation occurred both within the hospital’s actual clinical environment and in cyberspace. Spradley’s [23] definition of participant observation entails observing people’s activities, the physical attributes of the social context, and experiencing the scene as a participant. This term was chosen as 1 author actively participated in the entire research process, while the remaining 4 authors observed solely in cyberspace, utilizing the mobile messenger app Kakao Talk (Kakao Games) and the video communication platform Zoom (Zoom Video Communications). Consequently, the use of the term adequately aligned with Spradley’s approach.

### Participants

The selection of research participants and social situations followed the ethnographic research methodology [23,24] to accurately describe clinical nurses’ research environment and culture. Participants were purposefully selected based on factors that potentially influence research cultures, including position, research experience, education, and clinical experience. To attain a representation that resonates with the research culture of clinical nurses, recruitment focused on nurses with research experience, particularly those who had completed nursing research-related courses at a university hospital. Furthermore, as the research meetings were primarily conducted through Kakao Talk and Zoom, the inclusion criterion was the ability to use cell phones and computers.

Ethnography acknowledges that the required number of research participants varies depending on the cultural context [23]. Drawing from previous qualitative research [22] that focused on similar research topics and participants, a blend of purposive and snowball sampling strategies was used to recruit nurses engaged in nursing research in a hospital. The sample comprised 5 staff nurses and 1 nurse unit manager affiliated with the medical-surgical unit of a Korean tertiary hospital. One participant (who is a member of the hospital nursing research team and a contributing author to this ethnographic study) was actively involved in both participant observation and the ethnographic interview; this dual role allowed for close and continuous observation of the progress of unit-based nursing research from an actual internal perspective, enriching the study with insight from active engagement in research subjects.

### Data Collection

Data collection for this study was executed from April to May 2022, involving several methods, namely participant observation and ethnographic interviews. These diverse methodologies enabled researchers to garner rich data, obtain a deeper understanding of the cultural context, and address the study’s queries effectively.

#### Participant Observation

Participant observation encompassed interactions both within the actual clinical environment of the hospital and in web-based spaces during video research conferences. One of the authors, who was also a participant in the research, conducted in-depth observations, involving monitoring of the research process in the clinical setting and active involvement as a member of the hospital’s nursing research team. The remaining 4 authors observed remotely through video meetings on Kakao Talk and Zoom to oversee the research process.

**Figure 2:**
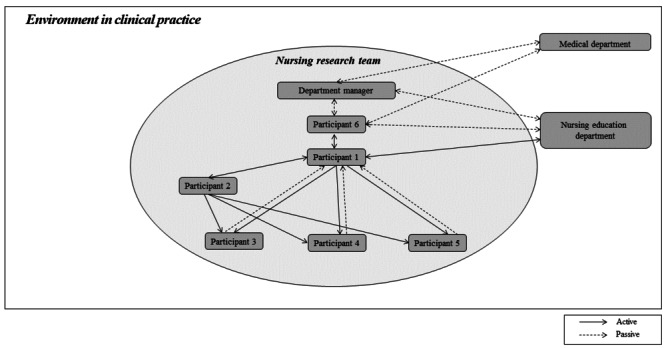
Interaction of the research participants.

The focus of both forms of participant observation was on noting participants’ activities and cultural and environmental characteristics, as well as identifying various aspects such as space, actors, activities, objects, behaviors, events, time, purpose, and emotions. The relationships between research participants, as identified through participant observation, are illustrated in Figure 2.

#### Ethnographic Interviews

Ethnographic interviews were conducted as one-on-one web-based sessions through Zoom in adherence to COVID-19 regulations. These interviews were facilitated by a single, experienced qualitative research interviewer who was not a part of the hospital nursing team. The interviews involved a mix of open-ended and semistructured questions, commencing with the following initial question designed to engage participants with the research topic: “What is the topic of your current hospital research?” Subsequent questions were aimed at eliciting in-depth, voluntary explanations from participants.

The structure and content of the interview questions and guidelines were informed by previous research on clinical nursing research in Korea [14,21,22,26] and were aligned with Spradley’s [24] ethnographic interview approach. Following the outlined interview guide (Multimedia Appendix 2), the interviews were conducted individually and typically lasted between 35 and 50 minutes, with the average duration being 40 minutes.

### Data Analysis

In this study, 5 authors acted independently as data coders, each coding the collected data. Discrepancies in the coding results were discussed in research meetings and subjected to a consensus process until an agreement was reached. Word (Microsoft Corp) and Excel (Microsoft Corp) were used for data analysis. Initial transcripts of reported activities were compiled in Word, and meaningful data related to the topic were identified and listed in Excel, with each sentence recorded in a separate row. Subsequently, related sentences were grouped to derive themes.

Data analysis was conducted iteratively alongside data collection, using Spradley’s [24] 4-step method consisting of domain analysis, taxonomic analysis, componential analysis, and theme analysis. In the domain analysis, we reviewed ethnographic interviews and transcripts of reported activities to identify meaningful domains related to the culture of clinical nursing research. These domains were categorized into six areas of clinical nursing research culture: (1) clinical application of nursing research, (2) research role assignment, (3) shift work, (4) hospital research resources, (5) interaction between researchers, and (6) purpose of nursing research.

Taxonomic analysis led to the construction of meaningful terms within the identified domains, resulting in 37 subcategories. Componential analysis distinguished the characteristics of terms used by participants in each classification, leading to the derivation of 12 categories. All authors revised and integrated these categorizations through meticulous review. Subsequent to the categorization and integration, we performed a theme analysis and selected the final meaningful data to provide insight into the culture of clinical nursing research. Contents with similar meanings were classified and categorized, revealing 6 subthemes related to the culture of the nursing research environment among clinical nurses. These subthemes were then synthesized into 3 overarching themes that offered a comprehensive and integrated understanding of the culture of nursing research in the clinical setting.

### Rigor

The rigor of this study was bolstered by using a variety of strategies recommended by Lincoln and Guba [27]. To ensure the accuracy of the interview content and methodology, the trained interviewer engaged in discussions with the other authors. All authors maintained transparency through critical reflection on their own beliefs, documented self-critical memos, and participation in deliberative discussions. Dependability was assured by integrating data collection and analysis in a simultaneous, cyclic approach. Additionally, a nursing professor well-versed in qualitative research continuously reviewed the processes of data collection and analysis to maintain the integrity of the study. Lastly, to assess their transferability, the findings were presented to other clinical nurses to gauge their applicability in varied settings.

### Ethical Considerations

This study received approval from the institutional review board of Ewha Womans University (202204-0002-01) and adhered to ethical guidelines. Potential participants were adequately informed about the study’s purpose, methods, and incentives, and voluntary participation was emphasized. Sufficient time was provided for potential participants to consider their involvement. Interested participants provided written informed consent and were assured of their right to withdraw from the study at any time. Participants were informed that the collected data would be used only for research purposes and that they could discontinue participation at any time during the study. Access to the collected data was restricted to the authors of the study. To maintain confidentiality, any identifying information and files that could link data to individual participants were securely discarded upon the completion of the study.

## Results

### Participant Characteristics

This study included 6 participants, all female, comprising 5 staff nurses and 1 unit manager from a ward. The participants were aged between 26 and 53 years and had clinical experience ranging from 2 to 30 years. The number of research experiences among the participants varied from 1 to 7 instances. Additional details on the participants’ characteristics are provided in Table 1.

The findings of the study are subsequently presented, supplemented by excerpts from the observations and interviews conducted with the participants.

**Table 1:** Demographic characteristics of the participants.

Number	Position	Number of research experiences	Education	Age (years)	Clinical experience (years)
1	Staff nurse	6	Doctoral student	34	6
2	Staff nurse	2	Master’s student	30	4
3	Staff nurse	2	BSN^a^	26	2
4	Staff nurse	1	BSN^a^	28	3
5	Staff nurse	1	BSN^a^	27	3
6	Unit manager	7	MSN^b^	53	30

^a^BSN: Bachelor of Science in Nursing.

^b^MSN: Master of Science in Nursing.

### Balancing Positive and Negative Influences in the Research Process

#### Shift Work and High Workload Negatively Impacting Research Progress

Nurses working in shifts and experiencing high workloads expressed feeling too exhausted to balance their work and research responsibilities. Most participants viewed research as a separate entity from their clinical roles and expressed that they found the research process arduous and challenging to juggle alongside their work duties. Nurses’ varying schedules resulting from shift work made finding a suitable meeting time challenging. Participants in this study used web-based meetings as a solution that allowed maximal participation, and they provided recordings for those who could not attend due to scheduling conflicts. Nonetheless, some participants found it difficult to discuss and share progress updates due to shifting work schedules and reported feeling too drained to attend research-related training sessions given their substantial workload. Consequently, the demands of high workloads and shift work often resulted in deprioritization and postponement of research activities.

The process of moving forward seems very arduous. Balancing work and research is challenging, and maintaining focus is difficult.

Participant 2, observation

Due to shift work, only a few nurses discuss and are informed about the progress of the research. This sometimes leaves others struggling to understand and keep up with the research's progress, which can be embarrassing.

Participant 3, interview

Sometimes, I feel so tired and overwhelmed by the high workload that I cannot afford to participate in research-related training.

Participant 4, interview

#### Positive Utilization of Hospital’s Research Support Resources

The research support resources provided by the hospital positively impacted the progress of the research. Nurses shared that they were able to submit clinical questions about which they were curious through a hospital program, leading to the formation of a research team and the initiation of research. The hospital provided various research support resources, such as research-related education and academic services, support for educational expenses, and dedicated human resources to assist with research. During meetings, participants referenced books provided by the hospital that contained essential information for advancing research. The accessibility of these resources cultivated a supportive environment that enabled participants to conduct more efficient and effective research, which could be translated into positive outcomes.

Every year, our hospital holds an event encouraging nurses to formulate research questions stemming from their clinical curiosities. I found myself jotting down sporadic thoughts, and these activities naturally evolved into nursing research.

Participant 4, interview

The Nursing Education Department collaborates with our research team leader, offering support including statistical consulting.

Participant 2, interview

Detailed information useful for assessing the “Risk of Bias” of the selected literature can be found on page 62 of the book provided by the hospital.

Participant 1, observation

### Fostering Transformational Change for Both Nurses and Patients

#### Selection of Research Topics Derived From Clinical Settings

Participants acknowledged the need for change to enhance the working environment for nurses and create a safer hospital environment for patients. They engaged with questions emerging from their daily practices and evolved these inquiries into research topics. They perceived that addressing these topics could trigger significant changes that would benefit both nurses and patients. This approach seemed to deepen their understanding of the prevalent issues and elevate the relevance of the research to clinical practice.

This research topic came about because nurses noticed issues while doing their jobs. They were thinking about other possible solutions since patients were having a hard time taking a lot of laxatives, causing them discomfort and making nursing tasks take longer.

Participant 6, interview

The issue we chose as our research topic was something I often pondered over during work. It was a mutual concern among all nurses and patients in our unit, and it’s intriguing to see it evolve into a research question.

Participant 4, interview

I firmly believe the clinical setting is the optimal environment for nursing research. Numerous topics are inherently connected to nursing practices and patient care, highlighting the immense value of conducting research in such settings. Given the chance, I aspire to continue pursuing research in clinical environments.

Participant 1, interview

#### Meaningful Outcomes Obtained Through the Research Progress

All participants regarded the knowledge obtained through research as a common, meaningful outcome, signifying that the acquisition of new knowledge was a significant and shared benefit experienced by the entire group. In addition to the shared benefit of knowledge, participants anticipated obtaining various individual benefits from their research process, including the development of leadership and followership skills, expertise in their field, tangible rewards, increased satisfaction, improved confidence, reinforced trust within the team, and a sense of group unity. The participants expressed that they enjoyed the research process and that the array of rewards it offered led to positive experiences for all involved.

Even if the results of our research don’t align with our hopes, I think our nurses have already grown personally during the process and can act as positive influences for our younger colleagues.

Participant 6, interview

I studied article search and analysis techniques in nursing school, but doing research in a clinical environment has allowed me to realize the importance of these skills firsthand, enhancing my learning confidence. I’m also thinking about attending graduate school, and I feel that my current research experience will be beneficial then.

Participant 5, interview

If our research is published in a scholarly journal, it would be a personal achievement, so I’m even more motivated to work harder.

Participant 2, interview

### Promoting Complementary Communication Among Nurses

#### Varied Research Participation Based on Research Competency

The level of involvement of nurses in the research varied, influenced by their previous research and postgraduate course experiences. This involvement was also correlated with the relationships among participants, as depicted in Figure 2. In essence, team members who were actively engaged in the research demonstrated more active relationships within the team, while those who were less active exhibited more passive relationships. Participants with research experience actively shared their opinions; however, as the research progressed, they felt the burden of the uneven distribution of tasks. Conversely, those without previous research experience performed only the roles assigned to them by their more experienced peers and felt apologetic toward other participants.

Having engaged in similar research during my master’s program, I find the current research less challenging. However, colleagues lacking research experience may find it somewhat hard to keep pace with the progress of the research.

Participant 2, interview

As the research becomes more complex, the team is finding it difficult, increasing my workload. I feel that if I don't keep at it, the research might halt, so I’m pushing through. Honestly, it’s somewhat overwhelming.

Participant 1, interview

My team leader assigns tasks to members. Since I lacked knowledge about research, my participation has been more passive. So, these days, when I observe some team members struggling with the research, I feel a profound sense of guilt. It’s challenging for me to decide what to do initially.

Participant 5, interview

#### Differences in Researcher Roles Depending on Research Participation

Participants’ roles in the research process were diversified, reflecting individual research capabilities and experience, which correlated with the level of their involvement in research. Those actively involved, particularly individuals with previous research experience or a master’s degree, autonomously delineated their roles, aligning them with the team members’ strengths and competencies. This strategy fostered a cooperative environment and optimized the unique skills of each member. Conversely, participants engaged more passively, typically those lacking research or a relevant educational background, conformed to the leaders’ opinions, and concentrated solely on assigned tasks, expressing that they found this approach to be less burdensome. To mitigate the disparities in research capabilities and experiences, participants maintained consistent meetings and endorsed reciprocal, complementary communication. Participants expressed that this emphasis on open dialogue and collaboration imbued them with a sense of preparedness to tackle challenges arising during the research process.

I believe that fostering learning and robust teamwork can simplify the research process. I often contemplate the optimal distribution of types and volumes of work, respecting individual researchers’ competencies and workload.

Participant 1, interview

I appreciate our task assignment approach. Given our shared workspace, we understand each other’s strengths, which, coupled with my professional and research commitments, makes focusing on my strengths less burdensome.

Participant 3, interview

Maintaining regular communication is pivotal. The nature of our shift work complicates assembling everyone for research meetings, but I am confident that persistent communication can deepen our understanding of individual roles in research and enable us to offset each other’s limitations.

Participant 5, interview

## Discussion

### Overview

**Figure 3:**
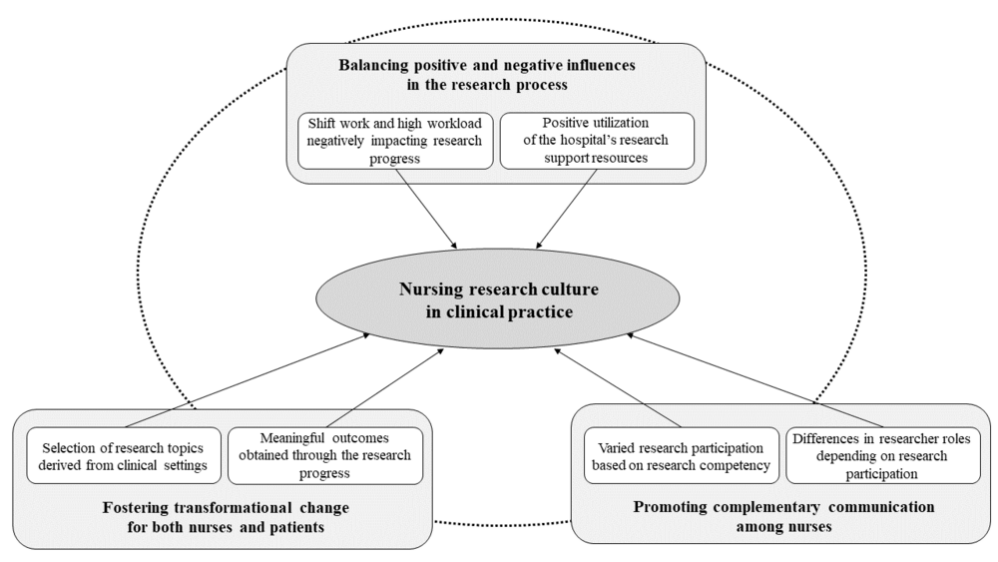
Essential themes of nursing research culture in clinical practice.

The findings of this qualitative study offer insights into the culture of nursing research in clinical settings, showcasing its potential to empower nurses to bring about positive transformations in patient care and their professional practice while bolstering collaborative efforts. The 3 identified themes are balancing positive and negative influences in the research process, fostering transformational change for both nurses and patients, and promoting complementary communication among nurses with different research competencies and roles. Figure 3 provides a visual representation of these themes. These findings underscore the crucial ability of nursing research to enhance nurses’ working environments, foster a safer atmosphere for patients, and facilitate overall progress and development in the clinical context.

### Principal Findings and Comparison With Previous Work

The first emergent theme was the balance between positive and negative influences in the research process. The clinical environment may serve as both a facilitator and a barrier for nurses conducting research. The availability of diverse research support resources plays a crucial role as a facilitator in enhancing clinical nurses’ research progress. A variety of research support resources can have a positive impact on clinical nursing research, including both material resources (eg, research-related education, academic services, and educational expense support) and human resources (eg, designated departments and personnel to assist with research progress) [28]. In Korea, clinical nurses have exhibited low research competency, a factor significantly correlated with the amount of organizational support [28]. Previous studies have indicated that organizational support and a strong belief in the value of clinical research enable research activity by fostering a culture that encourages the crucial exploration and application of research evidence in everyday practice [29]. Cultivating an organizational culture supportive of research at the institutional level is, therefore, essential to facilitating the clinical utilization of research findings.

Moreover, research participation by clinical nurses was observed to involve navigating between work and research commitments. Shift work creates challenges for scheduling regular research meetings on fixed dates and coordinating times when all research team members can gather. For example, a study in 2017 to describe the infrastructure supporting research in Magnet hospitals found that nearly half (44%) of the 249 hospitals responding required clinical nurses to conduct research activities during their regular clinical hours, and 40% reported nurses conducting research in their personal time; consequently, research activities often take a backseat to patient care priorities, making it challenging to allocate time for nurses away from direct patient care [30]. To encourage research by clinical nurses, dedicated time for research activities should be provided, and enhancements to the nursing working environment are imperative. Hospitals should acknowledge and account for the time invested in clinical nursing research within regular working hours.

The second theme underscored the transformative potential of clinical nursing research for both nurses and patients. Such research serves as a catalyst, allowing nurses to realize personal goals, such as enhancing their research capacity, while simultaneously fostering improved and safer environments for patients and health care providers. Consequently, deriving research questions from the clinical field and applying the research results to actual clinical practice is at the core of clinical nursing research [4].

In this study, participating nurses formulated research questions from their experiences caring for patients who had difficulty taking high-dose bowel cleansing solutions. Because clinical nursing research directly affects nurses’ work, specifically patient care, all our participants empathized deeply with the need for this research, and the practical applicability of the research results encouraged their active participation. The predominant themes identified in a previous study conducted with 64 perioperative nurses in a hospital in Korea (ie, learning how to solve problems in practice, facilitating team activities through motivation, barriers to large participation, and rewarded efforts and inflated expectations) [31] were congruent with the insights gained in this study, suggesting that to bolster clinical nursing research, it is essential to create opportunities for field-based question formulation and foster a belief in the capability to induce change. However, the urgency to partake in clinical nursing research should not overshadow the importance of undertaking thorough literature reviews on existing research findings related to clinical issues. Clinical nursing research should be pursued only when there is a paucity of evidence, and it must always adhere to ethical standards. Motivating nurses to engage in research, allowing for continual identification of pertinent research questions, and promoting thorough reviews of relevant existing literature can yield benefits for both nurses and patients and pave the way for research in previously unexplored areas.

The final theme revolves around complementary communication, accommodating the diverse competencies of nurses. The research team in this study encompassed nurses with varied research-related experiences. Differences in research competency among team members, attributable to varying levels of research experience, led them to adopt distinct approaches to research. Participants with extensive research experience had a better understanding of the research progress, which allowed them to take charge compared with those with less experience. Conversely, those with limited research exposure struggled with the unfamiliar content discussed in meetings and were uncertain about participation modalities. These findings are consistent with a previous study indicating that individuals lacking research experience or knowledge exhibit reluctance toward research participation [31]; therefore, research competency, inclusive of experience and knowledge, emerges as a pivotal facilitator in research implementation.

Despite the associated challenges, participants maintained complementary communication through regular web-based meetings to fulfill their research objectives. Successful complementary communication is straightforward, reciprocally advantageous, and reinforces continuous interaction and relationship development [32]. Given the evident benefits of such communication, we posit that fostering it within teams can significantly enhance nursing research in clinical settings. The diversity in research competency and roles among nurses highlighted in this study accentuates the necessity of nurturing complementary communication within research teams, thus ensuring equitable and balanced interactions and contributions among team members. In the research team examined in this study, the team leader allocated tasks, and nurses with less research experience assumed a more passive stance, fulfilling only the minimal tasks assigned. Communication was then leveraged to mitigate any arising discrepancies. We therefore suggest that championing complementary communication to address variances among research nurses while leveraging the individual strengths of nurses not only sustains clinical nursing research but also cultivates a positive research culture in clinical nursing.

### Limitations

This ethnographic study explored the nursing research culture in clinical nursing practice by examining the experiences of 6 nurses working at a tertiary hospital in Korea. The small sample size and the single-site setting may affect the transferability of the study’s findings, as they may not represent the broader population of clinical nurses. To mitigate this possibility, we amassed data until saturation was reached, with no additional information emerging. To bolster the study’s rigor, we shared the findings with nurses from various units and hospitals to assess transferability.

Due to the COVID-19 pandemic, participant meetings were held through Zoom, with scenes recorded for repeated review during analysis. This format hampered direct observation, however, limiting field notes to within-frame elements and omitting potentially significant out-of-frame expressions and movements. The shift to web-based methods challenges the traditional notion of “placeness of ethnography” [33], and some might argue that without physical immersion in the research area, there is no true fieldwork. However, digital platforms are enabling research in spaces where people are active, allowing a re-evaluation of the necessity of physical presence in traditional ethnographic fieldwork [34]. The paradigm that field research mandates physical colocation with participants [35] is undergoing reconsideration, especially given the COVID-19 pandemic, as technological advances redefine the concept of the research field [36].

### Conclusions

Clinical nursing research is pivotal in fostering nurse development and refining nursing practices by juxtaposing challenges such as intensive shifts and heightened workloads with facilitators such as patient-centric research questions and institutional research support. The clinical environment may serve dual roles as a facilitator by providing the requisite infrastructure for research and as a barrier when intensive shifts persist and research time is not allocated. Institutionalizing infrastructure for nursing research and earmarking time for such activities is crucial in clinical settings to facilitate continual knowledge circulation, thereby allowing nurses to generate and apply well-substantiated knowledge effectively. Adequate clinical nursing research enhances both professional development and patient care; therefore, nursing education programs should emphasize the importance of pinpointing apt research topics, reviewing existing research, and executing clinical nursing research. Subsequent research should probe whether the themes uncovered in this study accurately represent the nursing research culture in clinical settings and should identify the prerequisites for establishing an exemplary nursing research culture.

## Appendix

Multimedia Appendix 1 Standard for Reporting Qualitative Research (SRQR) checklist.

Multimedia Appendix 2 Interview Guide.

## Abbreviations

SRQR: Standards for Reporting Qualitative Research
