# Supplementary material for: Exploring Nursing Research Culture in Clinical Practice: Qualitative Ethnographic Study
Source: Asian Pac Isl Nurs J. 2024 Jan 9;8:e50703. doi: 10.2196/50703 (PMC10807379; doi:10.2196/50703)
Supplement: Multimedia Appendix 2 [file apinj_v8i1e50703_app2.docx]

**Appendix 2.** Interview Guide

| Title | Exploring nursing research culture in clinical practice: An ethnography approach |
| --- | --- |
| Aim | Exploration of the social and cultural context of research environment among clinical nurses in clinical settings |
| Research Questions | - What are the factors that promote research by clinical nurses in clinical settings?   What are the factors that hinder research by clinical nurses in clinical settings |

# Introduction

- Establish an interactive and comfortable atmosphere for the informant, ensuring confidentiality.
- Clearly communicate the purpose of the interview and emphasize the importance of obtaining an honest perspective from the informant.

| - By participating in this study, you will have the opportunity to reflect on your involvement in the research culture, which will serve as a basis for developing a clinical nursing research culture in the future. The data collected during the interview will be used for this purpose. - The interview will last approximately 60 minutes, and it will be recorded and noted from start to finish. You are encouraged to share your experiences and thoughts on clinical nursing research during the interview, and your honest perspectives will be highly valued. - If you have agreed to participate but prefer not to be recorded during the interview, please inform us, and we will discontinue the recording immediately. Your participation in this study is voluntary, and you can withdraw at any time without any negative consequences. The recorded file will only be accessible to the research members conducting the interview and will be analyzed with the researcher after notetaking. - The data collected from the interview will be kept strictly confidential and only accessible to the research team. It will not be used for any purpose other than this study, and your identity will be kept anonymous. |
| --- |

# Main

- Construct mandatory, technical, structural, and contrasting questions alternately.
- Do not raise objections to what the informant has mentioned, but let the interviewer know that he or she wants to know more through promoting questions ("Can you tell me more about that?" "I don't quite understand what you mean, can you explain it to me?").
- When I hear a 'taken-for-granted' statement (Lynd & Lynd, 1937), I ask the informant to help me understand it like I'm a fool so that I can understand it like others.
- If the conversation during the interview flows in an unhelpful direction for the purpose of the interview, make an effort to redirect it to the topic of interest.
- It is important to generate effective promoting questions or appropriate exploratory questions based on what happens during the interview.
  - Promoting questions: verbal/nonverbal signals used by the interviewer to keep the informant talking (leaning forward, making eye contact, nodding, making puzzled facial expressions, etc.).
  - Exploratory questions: questions used to supplement details, facilitate detailed explanations, and obtain clear explanations so that the informant can delve deeper into the topic ("When did that happen?" "Can you tell me more about that?" "I'm not sure if I understood what you meant." Be careful when asking 'why' questions, as they may have negative implications).
- Self-disclosure helps enhance mutual trust, but if the interviewer asks the informant to self-disclose during the interview, the informant may be manipulated or sensitive information may be leaked (Bogdan & Biklen, 1992).

| Types | Questions |
| --- | --- |
| General characteristics | Please provide your age, gender, education level, clinical experience, current hospital employment history, and frequency of research participation. |
| Descriptive | - Can you describe the process that led you to participate in the current research study? - What is your role in the current research study you are participating in? - To what extent do you feel that your intentions are reflected in the current research study you are participating in? |
| Descriptive /Structural | - Have you ever taken any education related to the research? If so, please let us know if the education helped you in performing the current research. - What were some of the difficulties you experienced during the research process? |
| Structural | - What was the most difficult thing you felt when conducting clinical research? - Do you have any motivation to continue research even though it is difficult and challenging? |
| Structural | - If there is something you want to gain by participating in the research, please let us know. - What helped you during the research process? Is there anything more you need? - What do you feel promotes nursing research? |
| Structural | - Do you intend to continue conducting clinical research in the future? If so, what is the reason? |

# Conclusion

- To give the sense that the conversation is coming to an end, provide a signal that the interview is almost over by saying something like "Before we finish, could you answer one more question?"
- Thank the informant for their time and acknowledge their importance to the interview by complimenting their honesty and insight.
- Express your gratitude for their participation and offer to follow up if any further information is necessary.

| - Thank you very much for taking the time to participate in this interview amidst your busy schedule. Your honest and insightful answers will be very helpful for our research. - If there are any parts of your narrative that need clarification, please feel free to request clarification. We can confirm if we understood your intentions correctly. - If additional explanation is needed for the interview content, we can conduct additional interviews via phone or email. The additional interview content will also be recorded and used for data analysis. - As a token of appreciation for your participation, we plan to provide a gift certificate (worth 10,000 Korean Won for observation and 30,000 Korean won for interview) after the observation or interview is completed. If additional interviews are conducted via email or phone, a gift certificate worth 5,000 Korean won per interview will be sent. |
| --- |
